# Mediating effects of mental and physical health on the association between chronic disease conditions and colorectal cancer screening utilization among breast cancer survivors

**DOI:** 10.1371/journal.pone.0328353

**Published:** 2025-08-06

**Authors:** Meng-Han Tsai, Jacqueline B. Vo, Justin X. Moore, Cody Ramin, Avirup Guha, Yanbin Dong

**Affiliations:** 1 Cancer Prevention, Control, & Population Health Program, Georgia Cancer Center, Augusta University, Augusta, Georgia, United States of America; 2 Georgia Prevention Institute, Augusta University, Augusta, Georgia, United States of America; 3 Division of Cancer Epidemiology & Genetics, National Cancer Institute, Bethesda, Maryland, United States of America; 4 Center for Health Equity Transformation, Department of Behavioral Science, Department of Internal Medicine, Markey Cancer Center, University of Kentucky College of Medicine, Lexington, Kentucky, United States of America; 5 Department of Biomedical Sciences, Cedars-Sinai Medical Center, Los Angeles, California, United States of America; 6 Cardio-Oncology Program, Georgia Cancer Center, Division of Cardiology, Department of Medicine: Cardiology, Medical College of Georgia at Augusta University, Augusta University, Augusta, Georgia, United States of America; Shuguang Hospital, CHINA

## Abstract

**Purpose:**

Breast cancer survivors have an increased risk of colorectal cancer (CRC) and those with chronic diseases are more likely to experience poor mental and physical health. For this study, we examined the mediating effects of mental and physical health on the association between chronic disease conditions and guideline-concordant colorectal cancer (CRC) screening among breast cancer survivors.

**Methods:**

We included 1,885 breast cancer survivors aged 45–75 years who were eligible for CRC screening in 2016, 2018, and 2020 Behavioral Risk Factor Surveillance System. The exposure was chronic diseases defined as prevalent diabetes, coronary heart disease/myocardial infarction, stroke, chronic obstructive pulmonary disease, emphysema/chronic bronchitis, arthritis, depressive disorders, or kidney diseases. The outcome was receipt of guideline-concordant CRC screening. Mediators were defined as self-reported frequent poor mental/physical health in the past 30 days (14–30 vs. 0–13 days). Multivariable logistic regression models were adjusted for sociodemographic and cancer-related factors. We used the methods proposed by Valeri & VanderWeele for the mediation analyses.

**Results:**

Breast cancer survivors with chronic diseases were 1.7-fold more likely to have CRC screening compared to those without any chronic diseases (OR, 1.68; 95% CI, 1.27–2.21). In mediation analysis, we found that frequent poor mental health mediated the association between chronic disease conditions and CRC screening utilization (−4.4% mediated; p-value = 0.035). We also observed a reduction through frequent poor physical health by 10.5% (p-value = 0.008).

**Conclusions:**

Frequent poor mental and physical health negatively mediated the association between the presence of chronic diseases and CRC screening utilization with a higher estimate for those with poor physical health. Effective implementation of integrated follow-up care is needed among breast cancer survivors to address chronic disease management and prioritize mental and physical health so that all patients receive guideline concordant CRC screening recommendations.

## Introduction

Advances in early detection and treatment have contributed to an increase in the 5-year survival rates for breast cancer which is now at 91% for all stages and 99% for early-stage diagnoses [1–3]. Breast cancer survivors however have an increased risk of colorectal cancer (CRC) [4] with a 2.5-fold increased risk compared to those without cancer [5]. The increased risk for CRC among breast cancer survivors may result from a combination of treatment toxicities (i.e., chemotherapy and radiation) [6], lifestyle factors, environmental exposures, and other shared etiological factors [7,8]. Accordingly, ensuring adherence to CRC screening recommendations remains crucial for breast cancer survivors [9]. Although there are several modalities for CRC screening [10,11], screening utilization among breast cancer survivors varies (46–82%) across study settings [9,12]. Factors associated with CRC screening uptake include sociodemographic factors [9,12], access to care (e.g., having a health care provider) [12], and utilization of survivorship care plans [13].

Breast cancer survivors, particularly those with chronic disease conditions, have increased healthcare utilization [14], which has the potential for improving adherence to CRC screening utilization [12]. However, breast cancer survivors with chronic disease conditions, such as heart failure [15], arthritis [16,17], and diabetes [16], have poorer physical and mental health outcomes compared to those without these conditions [15–18]. Poor physical and mental health can not only adversely affect survival outcomes [19,20] but may also decrease the likelihood of participating in CRC screening for preventive purposes among breast cancer survivors [21]. This may be particularly true for cancer survivors with chronic conditions, as they may be preoccupied with more immediate health concerns rather than other preventive care [22]. Consistent with our prior work, breast cancer survivors who had multiple chronic diseases (e.g., ≥ 3) and reported having frequent poor mental health were significantly less likely to be screened for CRC (OR=0.33, 95% CI = 0.14–0.74) compared to those with less frequent poor mental health and having multiple chronic diseases (e.g., ≥ 3) [23].

However, to date, limited studies have examined CRC screening adherence among breast cancer survivors with multiple chronic diseases [9,12,23], and none have assessed the mediating effects of frequent poor mental and physical health. Understanding these pathways can inform cancer survivorship care strategies among breast cancer survivors. Therefore, the current study aims to expand on prior research by evaluating two mechanisms. First, we examined the direct effect of chronic diseases on guideline-concordant CRC screening, hypothesizing that increased health care utilization may provide more opportunities for screening uptake. Second, we evaluated an indirect effect, where chronic diseases contribute to poorer mental and physical health, which in turn reduces the likelihood of CRC screening uptake among breast cancer survivors. This analysis was conducted using a nationally representative sample from the United States (US).

## Methods

### Study design and setting

For this study, we used the 2016, 2018, and 2020 Behavioral Risk Factor Surveillance System (BRFSS) data with available information on CRC screening utilization. The BRFSS is a large cross-sectional survey administered annually to approximately 400,000 adults aged ≥18 years across all 50 states, the District of Columbia, Guam, and Puerto Rico. The BRFSS methods and sample selection are described elsewhere [24]. Data on sociodemographic characteristics, healthcare access, health behaviors, and various chronic diseases, as well as cancer screening, were collected via questionnaires. Institutional Review Board (IRB) approval was granted by the respective health departments from each state and verbal consent was obtained with the BRFSS survey. BRFSS data is publicly available and de-identified and, therefore, exempt from IRB review at Augusta University.

### Study participants

**Fig 1:**
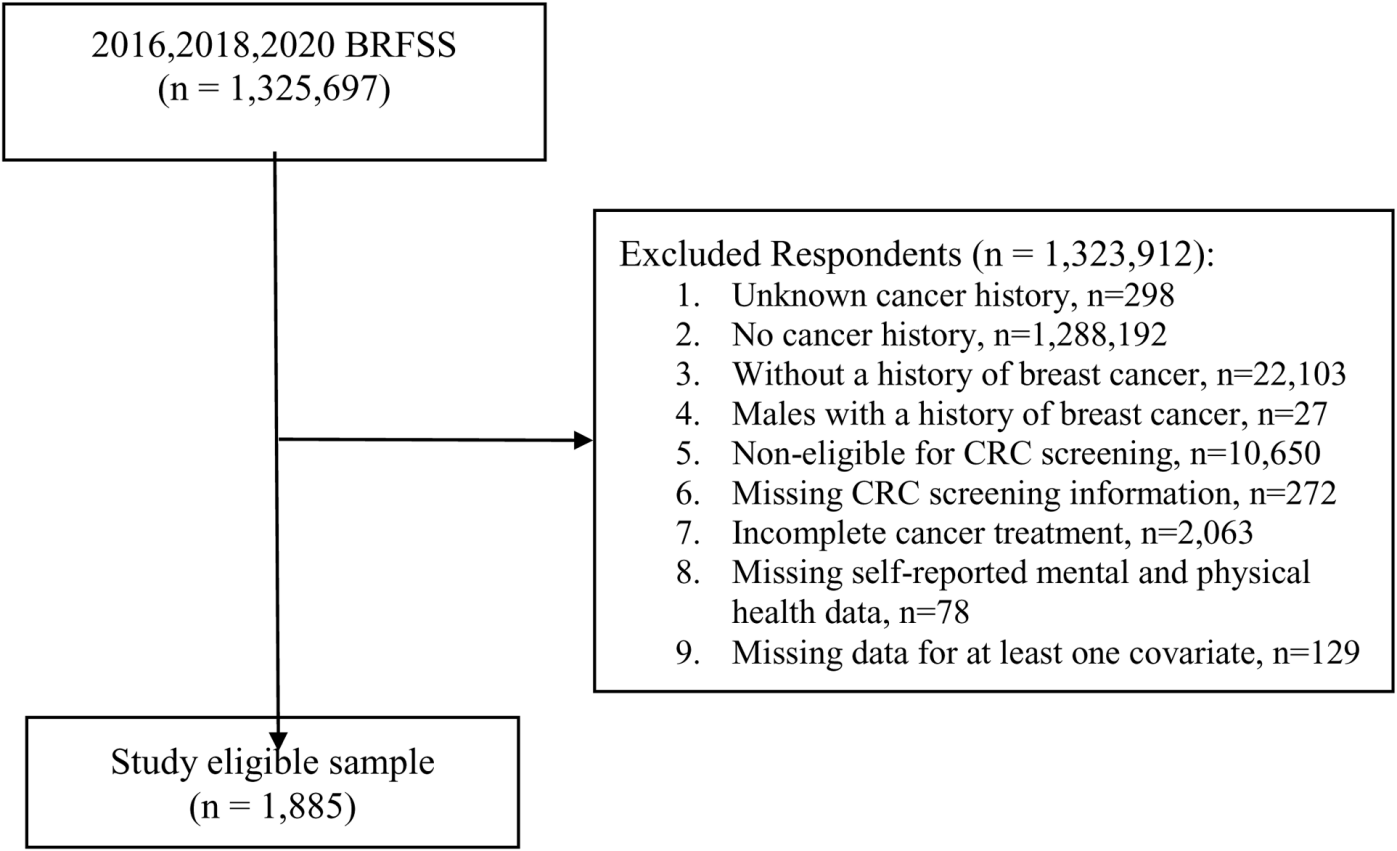
Study sample selection.

The 2016, 2018, and 2020 BRFSS data included 1,325,697 individuals aged ≥18 years. To select the eligible study sample, we excluded respondents with unknown cancer history (n = 298), without a history of cancer (n = 1,288,192), and without a history of breast cancer or CRC (n = 22,103). Males with a history of breast cancer were also not included in this study due to a small number of individuals (n = 27). Further, we limited our sample to survivors aged 45–75 years based on the CRC screening recommendations from the US Preventive Services Task Force (USPSTF) screening and the American Cancer Society (ACS) (n = 10,650 were excluded) [10,11] and those with CRC screening information (n = 272 were excluded). Women with incomplete cancer treatment were also excluded because the cancer survivorship module (e.g., whether receipt of follow-up care plans) in the BRFSS survey was only applied to survivors with complete treatment (n = 2,063). Finally, we excluded survivors with missing data on self-reported mental and physical health (n = 78). Other important covariates with missing information including race/ethnicity, education, current provider type, or receipt of follow-up care plans were also excluded (n = 129). As a result, we included 1,885 breast cancer survivors who have completed treatment as the study’s final sample (Fig 1).

### Exposure of interest: Chronic diseases conditions

Our primary exposure of interest was the presence of chronic disease conditions, which were self-reported data. These included diabetes, coronary heart disease (CHD) or myocardial infarction (MI), chronic obstructive pulmonary disease (COPD), stroke, emphysema or chronic bronchitis, arthritis, depressive disorders, or kidney diseases (not including kidney stones, bladder infection or incontinence). Chronic disease conditions were selected based on inclusion within the 2016,2018, and 2020 BRFSS surveys [25]. For this study, we categorized the presence of chronic disease conditions into a binary variable: (1) yes (having at least one disease) or (2) no (having no diseases). Outcome of interest: Guideline concordant CRC screening

Our primary outcome of interest was the receipt of guideline-concordant CRC screening (yes, no). Guideline concordant CRC screening was based on recommendations for average-risk individuals issued by the ACS and USPSTF [10,11]. Breast cancer survivors were defined as having guideline-concordant CRC screening if they reported receipt of 1) colonoscopy within 10 years, 2) sigmoidoscopy within 5 years, or 3) fecal occult blood test (FOBT) within 1 year. Survivors who never received CRC screening or those who did not receive a colonoscopy within 10 years, sigmoidoscopy within 5 years, or FOBT within 1 year were defined as not receiving guideline-concordant CRC screening.

### Mediators: Mental and physical health status

A mediator variable is a variable that changes the relationship between exposure of interest and outcome of interest and is considered to be along the pathway between exposure and outcome [26]. Therefore, breast cancer survivors’ self-reported mental and physical health status were considered as mediators for the association between chronic disease conditions and guideline-concordant CRC screening utilization in our study. Survivors’ mental and physical health status were classified using two BRFSS questions on how many days within the past 30 days participants experienced: 1) poor mental health, including stress, depression, and problems with emotions, and 2) poor physical health, including physical illness and injury. We categorized these two variables into binary variables defined as infrequent (0–13 days) and frequent (14–30 days) poor health using previously defined cut points [27].

### Covariates

Sociodemographic covariates included age (45–59, 60–69, or 70–74 years), race/ethnicity (non-Hispanic White, non-Hispanic Black, or Hispanic/Others (American Indian, Alaskan Native, Asian, Hispanic, others)), education (high school graduate or lower, some college graduate or higher), and annual household income (<$50,000, ≥ $50,000, or unknown). Cancer-related covariates included having insurance for cancer treatment (yes, no), current healthcare provider type (general practice or non-general practice), and follow-up care plan receipt (yes, no).

### Statistical analysis

We used logistic regression to estimate odds ratio (OR) and 95% confidence intervals (CIs) for the association between chronic disease conditions and guideline-concordant CRC screening utilization. We conducted four sequential models: model 1 included chronic disease conditions only, model 2 was additionally adjusted for sociodemographic characteristics, and model 3 was adjusted for all variables in model 2 plus cancer-related factors.

To examine the mediating roles of frequent poor mental and physical health on the association between chronic disease conditions and CRC screening utilization, we used the approach proposed by Valeri & VanderWeele (2013). This method allows for the decomposition of the total effect into two pathways. These pathways include 1) the direct effect, defined as the effect of the exposure on that outcome, not via the mediator, and 2) the indirect effect, defined as the effect of the exposure on the outcome via the mediator. This approach allows for the use of both binary outcomes and mediators [28,29]. With this approach, we can make valid inferences with the assumptions that the measured covariates are controlling for confounding between the 1) exposure and outcome (βa), 2) exposure and mediator (βb), 3) mediator and outcome (βc), and 4) that the mediator-outcome confounders are not affected by the exposure (βd) [28,29]. We presented results from mediation analysis as the 1) natural direct effects (NDE) (i.e., the effect of chronic disease conditions on CRC screening utilization), 2) natural indirect effect (NIE) (i.e., the effect of chronic disease conditions on CRC screening utilization through mental and physical health), 3) total effects (i.e., the total association between chronic disease conditions and CRC screening utilization), and 4) percent mediated with an indirect effect. Two sequential mediation models were performed, adjusting for sociodemographic characteristics (model M1) and sociodemographic plus cancer-related factors (model M2). The direct, indirect, and total effects were presented as the ORs and the corresponding 95% CIs. Two-sided p-values of <0.05 were considered statistically significant. We used SAS Version 9.4 (SAS Institute Inc., Cary, North Carolina) to perform all the analyses.

## Results

### Characteristics of breast cancer survivors by CRC screening utilization

Breast cancer survivors with chronic diseases had a higher prevalence of CRC screening compared to those without any chronic diseases (84.7% vs. 78.9%;p-value = 0.002) (Table 1). Older (>60 years) and non-Hispanic Black breast cancer survivors were more likely to have CRC screening utilization (both p-values <0.001). Breast cancer survivors with treatment insurance also had greater CRC screening utilization compared to those without any insurance (83.4% vs. 73.2%; p-value = 0.006). However, those with frequent poor mental (76.3%) and physical (78.1%) health had lower CRC screening use compared to those with infrequent poor health (mental health: 83.5%; physical health: 83.7%) (p-value<0.05).

**Table 1:** Characteristics of breast cancer survivors by CRC screening utilization (n = 1,885).

	Total	CRC screening	CRC screening	P-value^c^
**(n = 1,885)**	**Yes**	**No**
	**(n = 1,561)**	**(n = 324)**
	**n(%)**			
**Chronic disease conditions**				0.002
No	617(32.7%)	487(78.9%)	130(21.1%)	
Yes	1268(67.3%)	1074(84.7%)	194(15.3%)	
**Sociodemographic characteristics**				
**Age**				<0.001
45-59	451(23.9%)	343(76.1%)	108(24.0%)	
60-69	896(47.5%)	754(84.2%)	142(15.9%)	
70-74	538(28.5%)	464(86.3%)	74(12.8%)	
**Race/Ethnicity**				<0.001
Non-Hispanic White	1578(83.7%)	1312(83.1%)	266(16.9%)	
Non-Hispanic Black	195(10.3%)	174(89.2%)	21(10.8%)	
Hispanic/Others^a^	112(5.9%)	75(67.0%)	37(33.0%)	
**Education**				0.117
High school or lower	504(26.7%)	406(80.6%)	98(19.4%)	
Some college or higher	1381(73.3%)	1155(83.6%)	226(16.4%)	
**Income**				0.055
Less than $50,000	717(38.0%)	576(80.3%)	141(19.7%)	
$50,000 or more	875(46.4%)	743(84.9%)	132(15.1%)	
Unknown	293(15.5%)	242(82.6%)	51(17.4%)	
**Cancer related factors**				
**Cancer treatment insurance**				
No	112(5.9%)	82(73.2%)	30(26.8%)	0.006
Yes	1773(94.1%)	1479(83.4%)	294(16.6%)	
**Current provider type**				0.060
Non- general practices^b^	603(32.0%)	485(80.4%)	118(19.6%)	
General practices	1282(68.0%)	1076(83.9%)	206(16.1%)	
**Follow-up care plan**				0.101
No	271(14.4%)	215(79.3%)	56(20.7%)	
Yes	1614(85.6%)	1346(83.4%)	268(16.6%)	
**Quality of life**				
**Mental health**				0.012
0–13 days	1695(89.9%)	1416(83.5%)	279(16.5%)	
14–30 days	190(10.1%)	145(76.3%)	45(23.7%)	
**Physical health**				0.020
0–13 days	1598(84.8%)	1337(83.7%)	261(16.3%)	
14–30 days	287(15.2%)	224(78.1%)	63(22.0%)	

Abbreviation: CRC, colorectal cancer.

^a^Hispanic/others include Asian, American Indian/Alaskan Native, and other race.

^b^Non-general practices include surgeon, oncology, urologists, and others.

^c^Chi-square test was used. Column percentage was used for total and row percentage was used for CRC screening utilization.

### Chronic diseases and guideline-concordant CRC screening utilization

In the unadjusted analysis (model 1), breast cancer survivors with chronic diseases were 49% more likely to be screened for CRC (OR, 1.49; 95% CI, 1.15–1.92), which persisted when adjusting for sociodemographic characteristics (OR, 1.58; 95% CI, 1.22–2.07) and cancer-related factors (OR, 1.56; 95% CI, 1.19–2.03) (Table 2).

**Table 2:** Association between chronic disease conditions and CRC screening use among breast cancer survivors.

	Model 1^a^		Model 2^b^		Model 3^c^	
	**OR (95% CI)**	**p-value**	**OR (95% CI)**	**p-value**	**OR (95% CI)**	**p-value**
**Chronic disease conditions**						
No	Reference	0.002	Reference	0.001	Reference	0.001
Yes	**1.49(1.15,1.92)**		**1.58(1.22,2.07)**		**1.56(1.19,2.03)**	

Abbreviation: CRC, colorectal cancer; OR, odds ratio.

Bold text indicates significance.

^a^Total effect: Chronic disease conditions.

^b^Adjusted for sociodemographic characteristics (age, race/ethnicity, education, and income).

^c^Adjusted for sociodemographic characteristics (age, race/ethnicity, education, and income) and cancer related factors (cancer treatment insurance, current provider type, and follow-up care plan).

### Mediating effects of frequent poor mental and physical health on guideline-concordant CRC screening utilization

**Fig 2:**
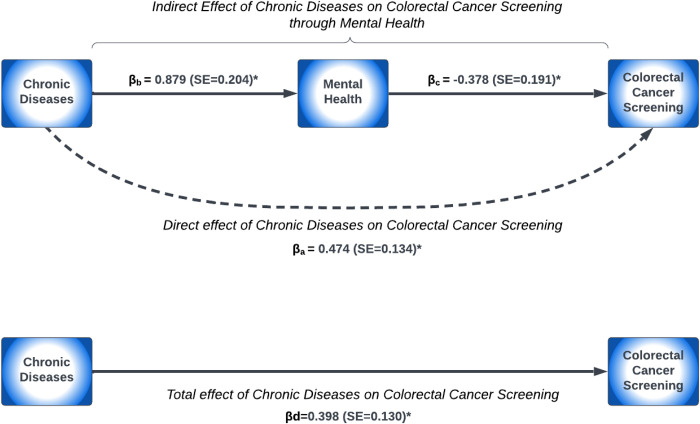
Paths from chronic diseases to mental health to CRC screening.

**Fig 3:**
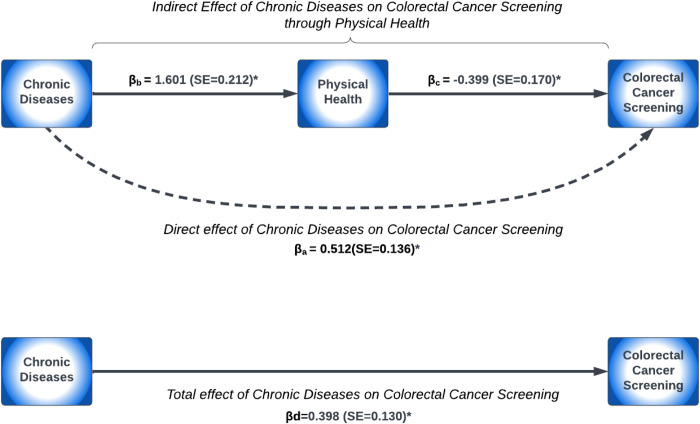
Paths from chronic diseases to physical health to CRC screening.

When adjusting for sociodemographic characteristics (model M1), survivors with frequent poor mental and physical health negatively mediated the effect of chronic disease conditions on CRC screening utilization (Table 3). Frequent poor mental and physical health was associated with a 4.3% and 10.1% reduction in the OR for CRC screening utilization, respectively. Similarly, after further adjusting for cancer-related factors (model M2), survivors with frequent poor mental and physical health had a 4.4% and 10.5% reduction in the OR for CRC screening utilization, respectively. A pathway diagram demonstrating the association between chronic disease conditions and CRC screening utilization mediated through frequent poor mental and physical health status adjusted for sociodemographic characteristics and cancer-related factors are presented in Figs 2 and 3, respectively. Survivors who had chronic diseases were 2.4-fold more likely to report frequent poor mental health (βb = 0.879; p-value <0.001), while those with frequent poor mental health were 32% less likely to have guideline-concordant CRC screening (βc = −0.378; p-value = 0.049) (Fig 2). In Fig 3, survivors who had chronic diseases were 5-fold more likely to experience frequent poor physical health (βb = 1.601; p-value <0.001), while survivors with frequent poor physical health had a 67% lower likelihood of CRC screening utilization (βc = −0.399; p-value = 0.019).

**Table 3:** Mediating analysis of mental and physical health on the association between chronic disease conditions and CRC screening use among breast cancer survivors.

	NDE	NIE	Total effect	Percent Mediated (%)^c^	p-value for NIE
**OR (95% CI)**	**OR (95% CI)**	**OR (95% CI)**
**Model M1** ^a^					
Mental health	1.64(1.26,2.12)	0.98(0.96,0.99)	1.60(1.24,2.08)	−4.3%	0.038
Physical health	1.70(1.30,2.22)	0.95(0.92,0.99)	1.62(1.25,2.10)	−10.1%	0.010
**Model M2** ^b^					
Mental health	1.61(1.24, 2.09)	0.98(0.96,0.99)	1.57(1.21,2.04)	−4.4%	0.035
Physical health	1.67(1.28,2.18)	0.95(0.92,0.99)	1.59(1.22,2.06)	−10.5%	0.008

Abbreviation: CRC, colorectal cancer; OR, odds ratio; NDE, natural direct effect; NIE, natural indirect effect.

^a^Adjusted for sociodemographic characteristics (age, race/ethnicity, education, and income).

^b^Adjusted for sociodemographic characteristics (age, race/ethnicity, education, and income) and cancer related factors (cancer treatment insurance, current provider type, and follow-up care plan).

^c^Percent mediated with indirect effect.

## Discussion

This study suggests that the association between chronic disease conditions and guideline-concordant CRC screening utilization among breast cancer survivors is influenced by the presence of frequent poor physical and mental health. Specifically, the mediating effects of frequent poor physical and mental health led to a notable reduction of 4–10% in CRC screening utilization among breast cancer survivors. This study represents the first exploration of potential mediation and sheds light on the complex interplay between chronic disease, health status, and cancer screening behavior among breast cancer survivors.

Consistent with prior studies, we also observed that breast cancer survivors with at least one chronic disease were more likely to have higher CRC screening use than those without chronic diseases (84.7% vs. 78.9%) without considering mental and physical health conditions. Our recent study using 2020 BRFSS data found that 92.3% of breast cancer survivors with ≥3 chronic disease conditions had greater CRC screening utilization [12]. Similarly, Dash and colleagues reported that breast cancer survivors with hypertension were two-fold more likely to have a colonoscopy compared to those without hypertension in a single center in Southern Maryland [9]; however, overall CRC screening rates were lower in this study compared to BRFSS respondents, potentially due to a higher proportion of insured BRFSS respondents which may lead to greater primary care access and CRC screening utilization. Dash and colleagues found similar results for colonoscopy utilization among breast cancer survivors with diabetes [9]. Although chronic disease conditions are not recognized as a strong determinant of guideline-concordant CRC screening in the general population [30], these findings indicate chronic disease conditions are an important factor for breast cancer survivors. Given that chronic disease incidence also rises sharply with age [31], it is possible that breast cancer survivors, particularly those with chronic disease conditions, have medical surveillance bias because they may have more regular healthcare visits for multiple disease management. Our finding suggests that breast cancer survivors with chronic diseases are more likely to prioritize their health and adhere to CRC screening guidelines, potentially leading to earlier detection and better outcomes for CRC. Therefore, this underscores the importance of comprehensive care and ongoing support for breast cancer survivors, as well as the need for healthcare providers to address the unique challenges of this population. This increased engagement with the healthcare system may lead to better coordination of care, including adherence to screening guidelines for other cancers.

Breast cancer survivors with poor mental and/or physical health may be less likely to accept other preventive services due to complex health conditions [32]. Our mediation analysis found that the ORs for the association between the presence of chronic diseases and CRC screening utilization were reduced by 4% and 10% due to frequent poor mental and physical health when survivors had chronic diseases, respectively. This finding highlighted the impact of poor mental and physical health on CRC screening adherence despite having at least one chronic disease associated with greater likelihood of having screening for CRC. A possible explanation may be because breast cancer survivors with a higher comorbidity score had substantially poorer physical and mental health outcomes [33] that may lead to lower screening adherence. Further, breast cancer survivors may also be more likely to experience poor mental and physical health due to cancer metastasis or recurrence. These prior findings may involve indirect evidence on the mediating effects of mental and physical health and warrant further investigation into longitudinal studies. In particular, the mediating effect of physical health on the mentioned association was much higher (10%). Therefore, clinical implications to improve CRC screening utilization among breast cancer survivors include supportive care interventions focused on prioritizing the improvement of mental and physical health [34–36], especially for breast cancer survivors with chronic diseases. Further, there is a need for effective implementation of follow-up care for CRC screening that include chronic disease management with a focus on mental and physical health through patient navigation programs to facilitate navigating complex multidisciplinary care teams [37–40].

Our study had several limitations. First, we could not examine the temporal effects of chronic disease conditions and mental/ physical health status using a cross-sectional study design. Further, respondents with a history of CRC may frequently have follow-up CRC screening due to cancer recurrence, which may overestimate their screening use. However, we were unable to exclude those who had various histories of cancers since the BRFSS cancer survivorship module was to collect respondents’ most recent cancer type only. Third, participants self-reported chronic disease conditions which could lead to underreporting of medical conditions. Similarly, recall bias is possible with CRC screening responses. Further, we could not include follow-up time since the diagnosis of breast cancer and age at diagnosis due to unavailable information in BRFSS surveys. The impact of chronic disease conditions on CRC screening decisions, as well as mediating effects of mental and physical health, may differ across various phases of survivorship periods. For example, greater CRC screening utilization may be observed within the first 5 years after breast cancer diagnosis compared to later periods due to regular oncologist visits for follow-up care. Finally, breast cancer survivorship was retrospectively collected and would have, at best, preceded mental health and physical health self-reported measures. Thus, it is plausible that the temporality of mental and physical health lies between breast cancer survivorship and the future likelihood of CRC screening. Despite these limitations, this is the first study to directly examine the mediation of frequent poor mental and physical health on chronic disease conditions and CRC screening among breast cancer survivors’ utilization using a nationally representative sample.

## Conclusions

Breast cancer survivors with chronic diseases were more likely to have CRC screening utilization but this association was mediated by frequent poor mental and physical health with a 4–10% reduction in CRC screening utilization. Effective implementation of integrated follow-up care in cancer survivorship care to address chronic disease management and prioritize mental and physical health is necessary so that all patients receive guideline concordant CRC screening recommendations. Patient-level interventions (e.g., patient navigation programs) may improve CRC screening utilization while simultaneously considering the mental and physical health of breast cancer survivors. Additional longitudinal research is warranted.
